# CO_2_ insufflation versus air insufflation for endoscopic submucosal dissection: A meta-analysis of randomized controlled trials

**DOI:** 10.1371/journal.pone.0177909

**Published:** 2017-05-24

**Authors:** Xuan Li, Hao Dong, Yifeng Zhang, Guoxin Zhang

**Affiliations:** 1 Department of Gastroenterology, the First Affiliated Hospital of Nanjing Medical University, Nanjing, China; 2 Department of Gastroenterology, the First School of Clinical Medicine of Nanjing Medical University, Nanjing, China; 3 Department of Cardiology, the Second Hospital of Nanjing, Nanjing, China; University Hospital Llandough, UNITED KINGDOM

## Abstract

**Background:**

Carbon dioxide (CO_2_) insufflation is increasingly used for endoscopic submucosal dissection (ESD) owing to the faster absorption of CO_2_ as compared to that of air. Studies comparing CO_2_ insufflation and air insufflation have reported conflicting results.

**Objectives:**

This meta-analysis is aimed to assess the efficacy and safety of use of CO_2_ insufflation for ESD.

**Methods:**

Clinical trials of CO_2_ insufflation versus air insufflation for ESD were searched in PubMed, Embase, the Cochrane Library and Chinese Biomedical Literature Database. We performed a meta-analysis of all randomized controlled trials (RCTs).

**Results:**

Eleven studies which compared the use of CO_2_ insufflation and air insufflation, with a combined study population of 1026 patients, were included in the meta-analysis (n = 506 for CO_2_ insufflation; n = 522 for air insufflation). Abdominal pain and VAS scores at 6h and 24h post-procedure in the CO_2_ insufflation group were significantly lower than those in the air insufflation group, but not at 1h and 3h after ESD. The percentage of patients who experienced pain 1h and 24h post-procedure was obviously decreased. Use of CO_2_ insufflation was associated with lower VAS scores for abdominal distention at 1h after ESD, but not at 24h after ESD. However, no significant differences were observed with respect to postoperative transcutaneous partial pressure carbon dioxide (PtcCO_2_), arterial blood carbon dioxide partial pressure (PaCO_2_), oxygen saturation (SpO_2_%), abdominal circumference, hospital stay, white blood cell (WBC) counts, C-Reactive protein (CRP) level, dosage of sedatives used, incidence of dysphagia and other complications.

**Conclusion:**

Use of CO_2_ insufflation for ESD was safe and effective with regard to abdominal discomfort, procedure time, and the residual gas volume. However, there appeared no significant differences with respect to other parameters namely, PtcCO_2_, PaCO_2_, SpO_2_%, abdominal circumference, hospital stay, sedation dosage, complications, WBC, CRP, and dysphagia.

## Introduction

Endoscopic submucosal dissection (ESD) allows lesions to be dissected and resected directly along the submucosal (sm) layer with use of an electrosurgical knife. With rapid advances in endoscopic techniques, ESD has become an invaluable tool in the treatment of early neoplasms of the gastrointestinal tract, particularly for large lesions[1]. However, this procedure is time-consuming and requires special endoscopic experience. The incidence of complications such as perforations, hemorrhage, and abdominal discomfort has also increased as a direct result[2].

Insufflation is required to achieve adequate visualization during ESD. Postoperatively, the gas is not absorbed immediately and remains in the gastrointestinal tract, which can cause abdominal pain and distension. Carbon dioxide (CO_2_) is rapidly cleared from the small bowel and excreted through the lungs, thus allowing the bowel to deflate quickly[3]. The benefits of use of CO2 over air were first pointed out by Rogers [4]. It is generally believed that use of CO_2_ insufflation is associated with less severe pneumoperitoneum, abdominal pain and abdominal distension[5, 6]. Recent clinical studies have shown that CO_2_ insufflation for ESD is safe and effective; however, Maeda et al[7] observed no significant difference with respect to post-procedural abdominal pain or discomfort between the CO_2_ insufflation and air insufflation groups. In addition, two meta-analyses of studies which compared the use of CO_2_ insufflation versus air insufflation for gastrointestinal endoscopy and endoscopic retrograde cholangiopancreatography (ERCP) reinforced the advantages of CO_2_ insufflation (lower post-procedural pain and bowel distension); however, they did not find any advantage with respect to arterial blood CO_2_ partial pressure (PaCO_2_) and transcutaneous partial pressure CO_2_ (PtcCO_2_) levels[8, 9].

Since then, numerous randomized controlled trials (RCTs) have addressed the role of abdominal discomfort and CO_2_ variation; however, other advantages of use of CO_2_ insufflation have not been adequately investigated. Few RCTs have assessed comprehensive indicators, such as the dosage of sedative drugs, procedure time and incidence of complications. Additionally, the number of patients in these RCTs were largely inadequate. Although the superiority of CO_2_ insufflation with respect to postoperative abdominal discomfort is backed by strong evidence from RCTs, the safety and efficacy of CO_2_ insufflation for ESD treatment has not been assessed by a meta-analysis. We, therefore, sought to assess the safety and efficacy of use of CO_2_ insufflation in patients undergoing ESD by performing a meta-analysis.

## Method

### Literature search

A literature search for relevant studies was conducted on online databases, Pubmed, Excerpta Medica Database (EMBASE), The Cochrane Library, Science Citation Index Expanded, and Chinese Biomedical Literature Database (Sinomed). All studies published as of January 2017 were eligible for inclusion. No restriction was imposed with respect to the language of publication or type of article. The following free-text terms and MeSH terms were used to retrieve studies: “carbon dioxide,” “ESD,” and “air”. The search strategy is summarized in S1 Table.

### Study selection and data extraction

Only RCTs that compared the use of CO_2_ insufflation with that of insufflation for ESD were selected. Two reviewers (Li X & Dong H) independently summarized information and data from each study using a standardized format. Any disagreement over study selection was resolved by referring to the adjudicating senior author (Zhang GX). Duplicate articles were excluded with the use of the software package Endnote X4 (reference management software). The title and abstract of the selected articles were screened to exclude articles that did not qualify the inclusion criteria, followed by a full text review of all eligible articles. In the event of lack of original data in the article, the respective authors were contacted to request further information.

### Outcomes

The primary outcomes for this analysis were (i) visual analogue scale (VAS) score for pain; (ii) percentage of patients without pain at various time points after ESD; and (iii) PtcCO_2_ and PaCO_2_ levels at different time points after ESD. The secondary outcomes were: abdominal distention, abdominal circumference, oxygen saturation (SpO_2_%), total procedure time; average hospital stay; dose of sedative drugs; incidence of complications (pneumonia, hemorrhage, perforation, and emphysema); clinical course (anal exsufflation, dysphagia, residual gas, and Mallory-Weiss tear); laboratory examination (white blood cell count [WBC] and C Reactive Protein [CRP] level).

### Quality assessment

Two authors (Li X and Dong H) independently evaluated the methodological quality of the included studies with use of the ‘risk of bias’ assessment tool from the Cochrane Handbook for Systematic Reviews of Interventions[10]. The following aspects of the methodology were assessed: random sequence generation, concealment of allocation, blinding of subjects and personnel, blinding of outcome assessment, completeness of reporting of outcomes data, potential reporting bias, and other sources of bias. The studies were divided into three groups based on the assessed risk of bias: high risk of bias, low risk of bias, and unclear. Disagreements were resolved by referring to the third author (Zhang GX).

### Statistical analysis

Meta-analyses were conducted with Review Manager 5.3 (Cochrane Collaboration, Oxford, UK). The random-effects model was used for all analyses owing to clinical heterogeneity among the selected studies[11]. The outcome variables were dichotomous. Risk ratios (RR) with 95% confidence intervals (CIs) were calculated by Mantel-Haenszel method. For continuous variables, mean difference (MD) with 95% CIs were calculated with inverse variance method[12]. For continuous outcomes measured on different scales, standardized mean difference (SMD) with 95% CI is reported. Heterogeneity among the selected trials was assessed with *I*^*2*^ measure of inconsistency (cutoff level of *I*^*2*^ = 50%). Sensitivity analyses were performed only for high-quality studies. If sufficient data were available, publication bias was assessed with use of funnel plots.

## Results

### Identification and characteristics of studies

**Fig 1:**
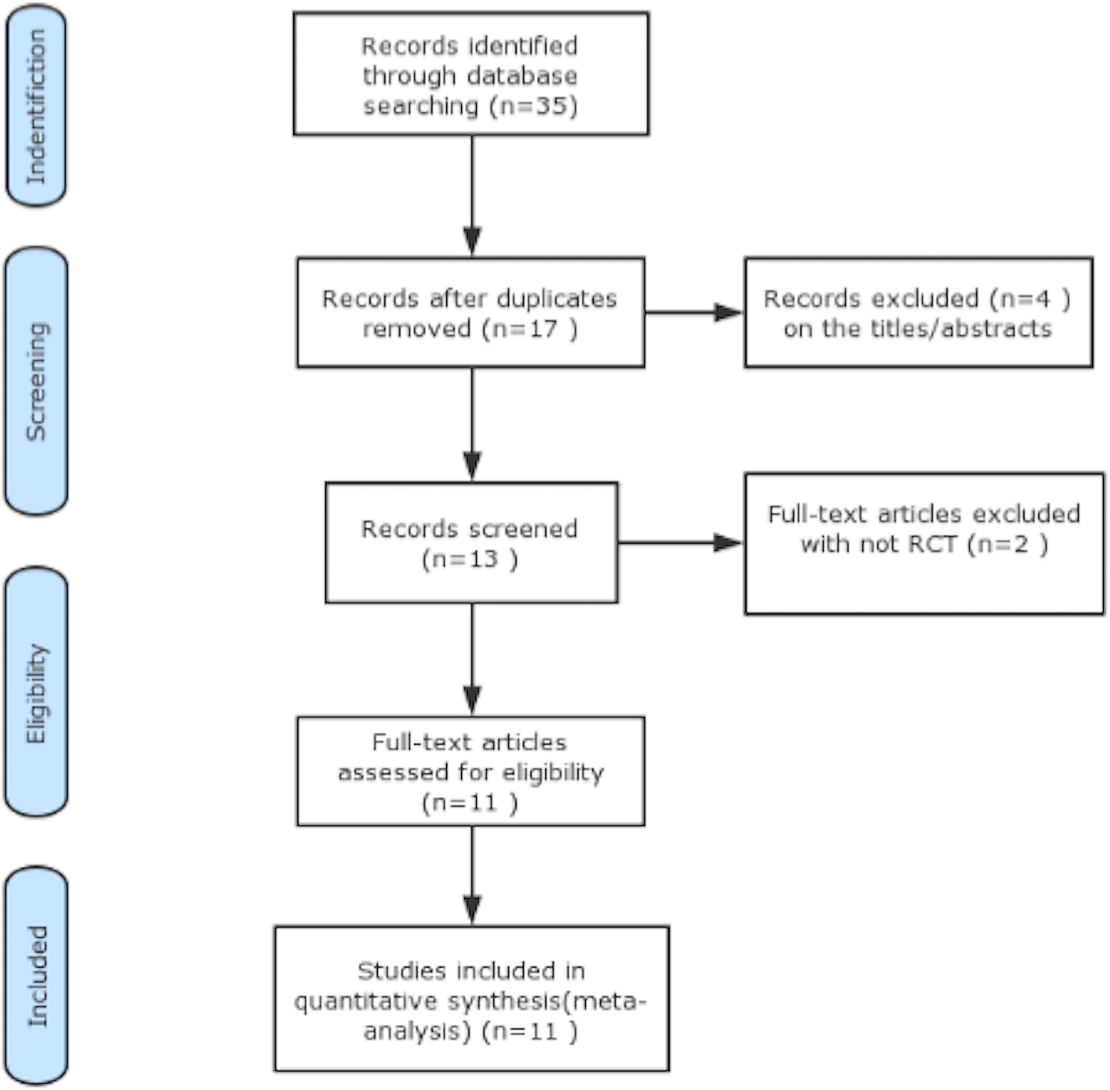
Flow diagram of studies identified, included, and excluded.

A total of 37 records were retrieved on initial search on electronic databases, including The Cochrane Library (*n* = 7), PubMed (*n* = 9), EMBASE (*n* = 12), Chinese Biomedical Literature Database (*n* = 6), and manual search of the references of the included RCTs (*n* = 1). Eighteen duplicates and four non-relevant articles were excluded after review of titles and abstracts. After a review of full texts, 2 articles with retrospective study design and an uncontrolled study were excluded. Finally, eleven publications were included in this meta-analysis; of these, ten were original research articles published in journals while one was a conference abstract. Fig 1 illustrates the process of literature search and study selection.

The characteristics of included studies are summarized in Table 1. A total of 1026 patients were involved; 506 were administered CO_2_ insufflation and 522 were administered air insufflation. In the third trial, 30 participants (12 from the CO_2_ group and 18 from the air group) particularly underwent further analysis, including PaCO_2_, procedure time, and sedative drugs dosage. All studies were conducted in Asia: eight in Japan[7, 13–18], three in China[19–21], and one in South Korea[22]. ESD equipment used in the trials included Olympus Optical Co[13, 15], Olympus GIF- Q260J[18, 21, 22], Olympus XGIF-2T240 M[7], Olympus 165[14], Pentex 2940[19], and Olympus CV-260SL[20]. For two articles, relevant details were not mentioned[16, 17].

**Table 1:** Characteristics of the included studies.

	Study	Recruitment period	Country	Design	Endoscope	Sample size (CO_2_/Air)	Mean age (CO_2_/Air)	Gender	
								Male	Female
								(CO_2_/Air)	(CO_2_/Air)
**1**	Nonaka et al[13]	Mar 2007 -Jul 2008	Japan	Prospective,RCT,Double-blind	Olympus Optical Co	89(45/44)	68.5±8.8/67.6±8.0	39/38	6/6
**2**	Maeda et al[7]	Feb 2011- Sep 2011	Japan	Prospective,RCT,Double-blind	Olympus XGIF-2T240 M	102(54/48)	72.5±9.0/72.0±10.2	40/35	14/13
**3**	Takada et al[23]	Jan 2009 -Dec 2009	Japan	Prospective,RCT,Double-blind	Olympus 165	87(36/51)	74.0±8.7/70.0±12.0	22/36	14/15
**4**	X Liu et al[19]	Jan 2013 -Dec 2014	China	Prospective,RCT,Double-blind	Pentex 2940	80(40/40)	58.4±10.8/57.2±12.6	21/25	19/15
**5**	L Zhan et al[20]	Jan 2012- May 2014	China	Prospective,RCT,Double-blind	Olympus CV-260SL	158(75/83)	39.6±7.1 /40.2±6.5	40/45	35/38
**6**	HK Feng et al[21]	May 2011- Mar 2013	China	Prospective,RCT,Double-blind	Olympus GIF—260j	97(56/41)	Not reported	Not reported	Not reported
**7**	Kim et al[22]	May 2012-Aug 2014	Koran	Prospective,RCT,Double-blind	Olympus GIF- Q260J	102(50/52)	81.8±9.5/62.0±7.5	34/16	38/14
**8**	Saito et al[15]	Nov 2004- May 2005	Japan	Prospective,RCT,Double-blind	Olympus Optical Co	70(35/35)	Not reported	Not reported	Not reported
**9**	Mosby et al[16]	Not reported	Japan	Prospective,RCT,Double-blind	Not reported	110(55/55)	Not reported	Not reported	Not reported
**10**	Onogi et al[17]	Jan 2009- Dec 2009	Japan	Prospective,RCT,Double-blind	Not reported	87(36/51)	Not reported	Not reported	Not reported
**11**	Maeda et al[18]	Feb 2011-May 2012	Japan	Prospective,RCT,Double-blind	Olympus GIF-Q260J	46(24/22)	67.5±5.8 /72.0±7.2	21/3	19/3

### Risk of bias assessment

**Fig 2:**
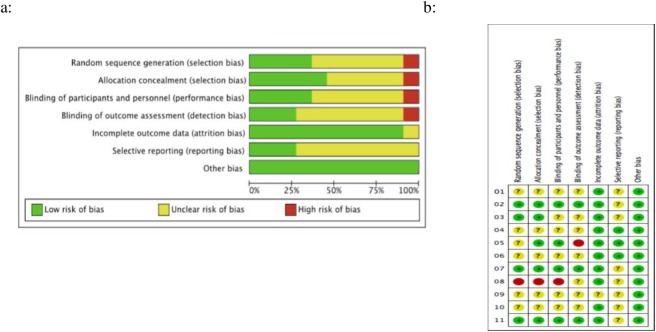
Results of quality assessment by Cochrane risk of bias. a. each risk of bias item presented as percentages across all included studies. b. each risk of bias item for each included study.

Cochrane risk of bias summary is shown in Fig 2. In six RCTs, group allocation was based on computer generated random number sequence or with use of an opaque envelope[7, 14, 16, 18, 20, 22]; for four RCTs, details of allocation concealment methods were not reported in adequate detail[13, 17, 19, 21]. With regard to information bias, both the participants and the endoscopists were blinded to the type of gas used[7, 13, 14, 16, 18–22], with the exception of the eighth and the tenth study[15, 17]. Immediate post-procedure assessment was performed by an assistant blinded to group allocation[7, 14–16, 19–22], with the exception of the tenth study[17]. In the seventh study, 8 patients who did not complete questionnaires were excluded from the final analyses[22]. For the other trials rated as ‘low risk of bias’ had no participant loss [7, 13–17, 19–21]. Most studies included were rated as ‘high risk of bias’[13–18, 20, 21], because of selective reporting of outcomes.

### Outcome measures

Data on PtcCO_2_ levels[7, 13, 14, 17, 18], PaCO_2_ levels[14, 15, 19–21] was reported by five studies each. Data on SpO_2_% after ESD was reported for four studies [7, 13, 14, 18]. The 100 mm VAS was used to grade abdominal pain[7, 16, 18–20] and abdominal distention[20, 22]. The scale ranges from 0 (no pain) to 100 (maximal pain)[24]. The percentage of patients with abdominal pain was also evaluated[7, 21, 22]. Only three studies reported data on median hospital stay[7, 14, 21]. Data on operation time measured from the start of circumferential marking to the completion of resection was reported for six studies[7, 14–16, 18, 21]. For seven trials, data on use of sedatives was reported (propofol, midazolam, and morphine)[7, 13–15, 18, 20, 22]. Complications (Perforation, haemorrhage, pneumonia, and emphysema) associated with the procedure were reported for six studies[14, 15, 19–22]. Data on WBC counts and CRP levels were reported in the sixth and seventh studies[7, 14]. Three articles assessed the clinical course (dysphagia, residual gas, and Mallory-Weiss tear) in the two groups after treatment[7, 14, 18].

#### 1.1 Primary outcomes: Abdominal pain

**Fig 3:**
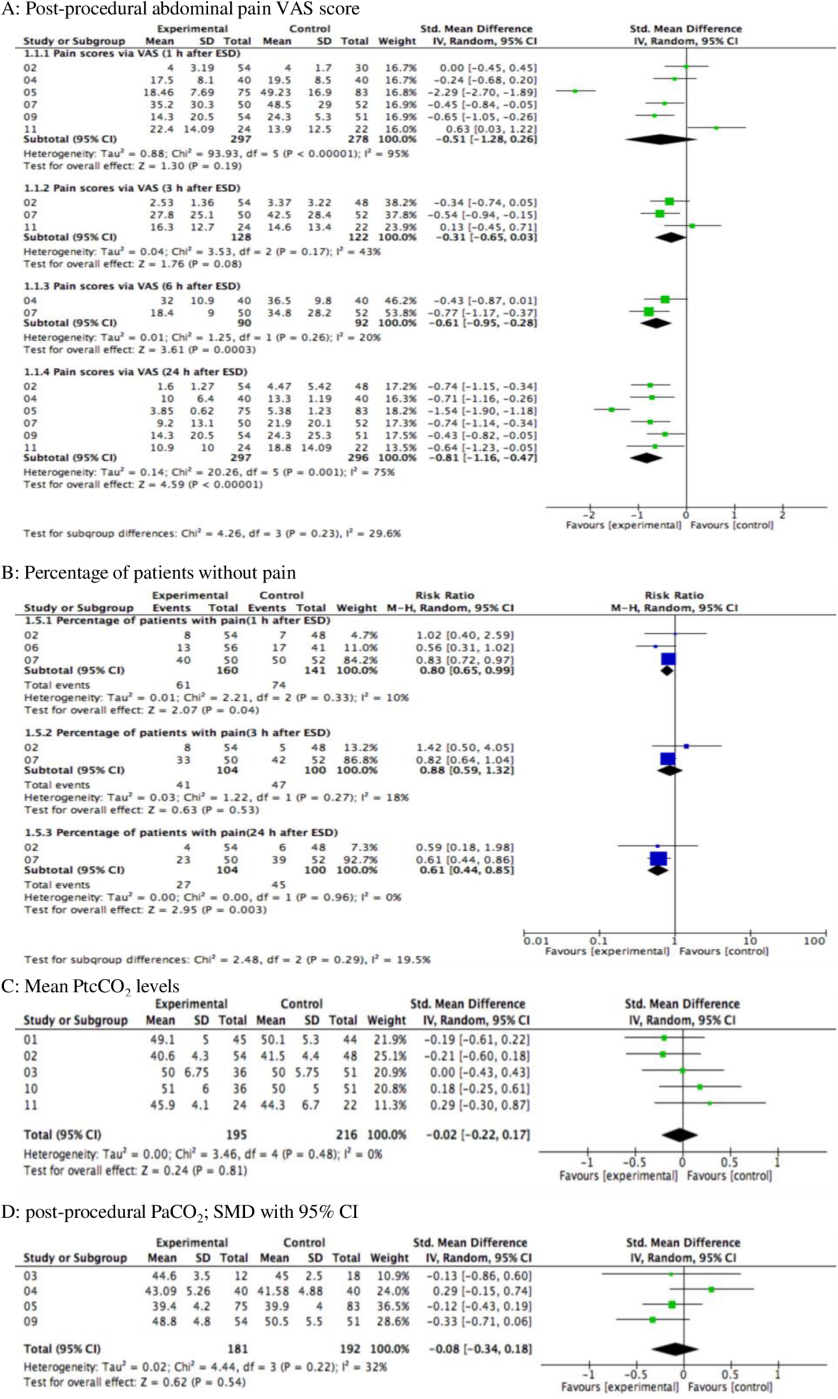
Forest plot of primary outcomes of ESD with CO_2_ insufflation and air insufflation. A: post-procedural abdominal pain VAS score; SMD with 95% CI; B: Percentage of patients without pain; RR with 95% CI; C: mean PtcCO_2_ levels; SMD with 95% CI; D: post-procedural PaCO_2_; SMD with 95% CI.

Pain VAS score and percentage of patients without pain was recorded at the following time-points: 1 h, 3 h, 6 h, and 24 h after ESD. Subgroup analyses was performed to assess these two outcomes. (i)Pain VAS score: Six articles reported pain VAS scores at 1 h and 24 h after ESD[7, 16, 18–20, 22]; three articles reported VAS scores at 3h and 6h after ESD[7, 16, 18]. The meta-analysis revealed that pain VAS scores at 6h and 24h post-procedure in the CO_2_ insufflation group were significantly lower than those in the air insufflation group (*P =* 0.0003 and *P<*0.00001, respectively). The difference in VAS scores at 1h and 3h after ESD were not statistically significant **(**Fig 3A). (ii)Percentage of patients without pain: Maeda *et* al[7]and Kim *et* al[22] reported the post-procedural pain experience as percentage of patients who did not experience pain at 1h, 3h, and 24h after ESD. Feng *et* al[21]only reported the rate of pain-free patients at 1h post-procedure. No significant higher proportion of patients was observed in the air insufflation group at 3h after ESD; however, the number of patients with pain was significantly smaller in CO_2_ insufflation group at 1h and 24h after ESD (Fig 3B).

#### 1.2 Primary outcomes: PtcCO_2_

Mean PtcCO_2_ levels after ESD were reported in five RCTs[7, 13, 14, 17, 18]. Meta-analysis showed no significant between-group difference in this respect (SMD = -0.02, 95%CI:-0.22–0.17, *P* = 0.81; *I*^*2*^ = 0%; Fig 3C).

#### 1.3 Primary outcomes: PaCO_2_

Mean PaCO_2_ levels after ESD were reported in four articles[14, 19–21]. Use of CO_2_ insufflation was not associated with a significant decrease in postoperative PaCO_2_ (SMD = -0.08, 95%CI:-0.34–0.18; Fig 3D). There was no significant heterogeneity among these four articles (*I*^*2*^ = 32%, *P* = 0.54). In general, the trend was consistent with that of mean PtcCO_2_ levels after ESD.

#### 2.1 Secondary outcomes: Abdominal distention VAS score

**Fig 4:**
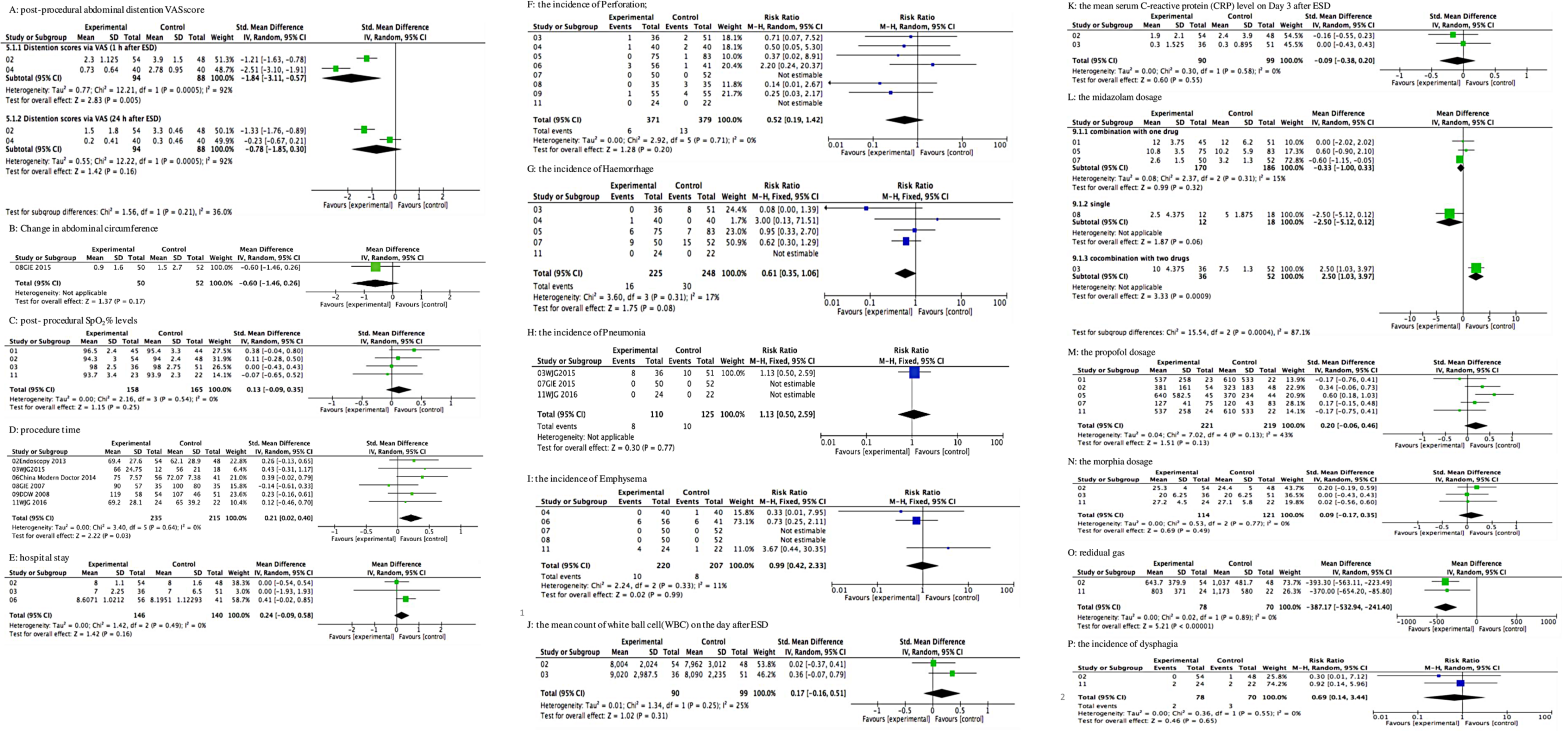
Forest plot of secondary outcomes with CO2 insufflation group and air insufflation for ESD. A: post-procedural abdominal distention VAS score; SMD with 95%CI; B: Change in abdominal circumference; SMD with 95%CI; C: post-procedural SpO2% levels; SMD with 95%CI; D: Procedure time; SMD with 95%CI; E: hospital stay; SMD with 95%CI; F: the incidence of Perforation; RR with 95%CI; G: the incidence of Haemorrhage; RR with 95%CI; H: the incidence of Pneumonia; RR with 95%CI; I: the incidence of Emphysema; RR with 95%CI; J: the mean count of white ball cell(WBC) on the day after ESD; SMD with 95%CI; K: the mean serum C-reactive protein (CRP) level on Day 3 after ESD; SMD with 95%CI; L: the midazolam dosage; SMD with 95%CI; M: the propofol dosage; SMD with 95%CI; N: the morphia dosage: SMD with 95%CI; O: redidual gas; MD with 95%CI; P: the incidence of dysphagia; OR with 95%CI.

Two studies utilized VAS score to assess abdominal distention at 1h and 24h post-procedure[7, 19]. VAS scores at 1h and 24 h after ESD were higher in the air insufflation group. The between-group difference at 1h after ESD was statistically significant (SMD = -1.84, 95%CI: -3.11- -0.57, *P* = 0.005; *I*^*2*^ = 92%; Fig 4A), while that at 24h after ESD was not statistically significant (SMD = -0.78, 95%CI: -1.85–0.30, *P* = 0.16; *I*^*2*^ = 92%; Fig 4A).

#### 2.2 Secondary outcomes: Abdominal circumference

Just one study reported the change in abdominal circumference after ESD[22]. Abdominal circumference after ESD was lower in the CO_2_ group, though the difference was not statistically significant (SMD = -0.60, 95%CI:-1.46–0.26, *P* = 0.17; Fig 4B).

#### 2.3 Secondary outcomes: SpO2%

Postoperative SpO_2_% after ESD was also reported by 4 studies[7, 13, 14, 18]. No significant difference in SpO_2_% was observed between the two groups (SMD = 0.13, 95%CI:-0.09–0.35, *P* = 0.25; *I*^*2*^ = 0%; Fig 4C).

#### 2.4 Secondary outcomes: Procedure time and hospital stay

With regard to procedure time, all of the air group procedures were completed without delay[7, 14–16, 18, 21]. However, use of CO_2_ insufflation was associated with significantly shorter procedure time (SMD = 0.21, 95% CI: 0.02–0.40, *P* = 0.03; *I*^*2*^ = 0%; Fig 4D). None of patients required extended hospitalization[7, 14, 21]. No significant between-group difference was observed with respect to the length of hospital stay (Fig 4E).

#### 2.5 Secondary outcomes: Complications

Perforation, haemorrhage, pneumonia, and emphysema were the main complications in seven studies. The four complications appeared to be balanced between the CO_2_ insufflation and the air insufflation groups[14, 15, 18–22]. No significant heterogeneity was observed among these studies (Fig 4F–4I).

#### 2.6 Secondary outcomes: Laboratory examination

No significant between-group difference was observed with respect to WBC counts and CRP levels on day-1 after procedure in two studies [14] (Fig 4J and 4K).

#### 2.7 Secondary outcomes: Sedative drugs

Seven articles reported the dosage of sedative drugs[7, 13–15, 18, 20, 22], which included propofol, midazolam, and morphia. Only Saito *et* al[15] used midazolam to maintain the depth of sedation, while Takada *et* al[14] used midazolam in combination with diazepam and pentazocine; the others[13, 18, 20, 22]were all combined other drugs to general anesthesia. Compared to air insufflation, dosage of sedative drugs did not obviously decrease in CO_2_ insufflation group, except when midazolam was used alone (Fig 4L–4N).

#### 2.8 Secondary outcomes: Clinical course

The included studies reported the following clinical parameters: residual gas, dysphagia, and Mallory-Weiss tear. Maeda *et* al reported dysphagia and gas volume in the gastrointestinal tract[7, 18]. The residual gas volume in the CO_2_ insufflation group was significantly lower than that in the air insufflations group (*P*< 0.00001, Fig 4O); dysphagia occurred in three patients in the air group and two patients in the CO_2_ group (Fig 4P). Mallory-Weiss tear was described by Takada *et* al [14] and Onogi *et* al[17]; the incidence of Mallory-Weiss tear in the CO_2_ group was significantly lower than that in the air group (0% *vs*. 15.6%, *P* = 0.013)[14]. Onogi *et* al[17] also reported lower incidence with use of CO_2_ insufflation; however, specific data has not been reported.

### Subgroup analysis and sensitivity analysis

As for heterogeneity with abdominal pain, abdominal distention and dosage of midazolam, we performed analysis of subgroup on the basis of a combination of drugs, country and type of Endoscope. However, the heterogeneity could not totally eliminate. It may be due to the sedation degree, the skill of endoscopist, etc. We could not conduct subgroup analyses on them as lack of original data. Only two studies[13, 14] reported abdominal distention, which hardly to accomplish the subgroup analysis.

On sensitivity analysis with regard to abdominal pain VAS scores at 1h and 24h post-procedure, heterogeneity decreased slightly after exclusion of the data from the study by L Zhan et al[20] (Tables 2 and 3). It can be speculated that an assistant who was not blinded to the gas being used may have been involved in the outcomes assessment.

**Table 2:** Changes in the heterogeneity of abdominal pain VAS score at 1h post-procedure after sequential exclusion of one study at a time.

	Excepted Data	Heterogeneity (*I*^2^%)	Increase or decrease rate
**2**	Maeda et al[7]	95%	0%
**4**	X Liu et al[19]	96%	1%
**5**	L Zhan et al[20]	73%	-23%
**7**	Kim et al[22]	96%	1%
**9**	Mosby et al[16]	96%	1%
**11**	Maeda et al[18]	95%	0%

**Table 3:** Changes in the heterogeneity of abdominal pain VAS score at 24h post-procedure after sequential exclusion of one study at a time.

	Excepted Data	Heterogeneity (*I*^2^%)	Increase or decrease rate
**2**	Maeda et al[7]	80%	5%
**4**	X Liu et al[19]	80%	5%
**5**	L Zhan et al[20]	0%	-75%
**7**	Kim at el[22]	80%	5%
**9**	Mosby et al[16]	73%	-2%
**11**	Maeda et al[18]	80%	5%

## Discussion

In this meta-analysis, we assessed the safety and efficacy of CO_2_ insufflation versus that of air insufflation for ESD treatment. The key finding of our analysis is that use of CO_2_ insufflation was associated with lower abdominal pain and distension in the postoperative period. Moreover, use of CO_2_ insufflation was associated with shorter procedure time. Finally, use of CO_2_ insufflation offered a distinct advantage of lower residual gas volume in the gastrointestinal tract. However, no advantage of CO_2_ insufflation was observed with respect to CO_2_ retention, SpO_2_%, the length of hospital stay and abdominal circumference. Overall, this meta-analysis suggests that CO_2_ insufflation is a safe and effective alternative to use of air insufflation for ESD.

We mainly assessed the extent of post-procedural abdominal discomfort and CO_2_ retention. Gas is deliberately insufflated into the gut lumen during ESD to facilitate visualization. In comparison with air, the gastrointestinal mucosa absorbs CO_2_ faster, which is subsequently eliminated via the lung. Therefore, CO_2_ insufflation possibly decreases the duration of abdominal distension[25]. However, Maeda *et* al[7] revealed that CO_2_ insufflation was irrelevant to the subjective pain and distension of patients. An RCT[14] showed no effect on the number of patients without pain. The results of our meta-analysis suggest that CO_2_ insufflation alleviated abdominal pain for at least 6h, and abdominal distension at 1h. Moreover, the percentage of patients without pain was also decreased, although the number of included RCTs was less. Whether CO_2_ insufflation induces metabolic disorder such as CO_2_ retention and decrease in SpO_2_% remains to be clarified. Actually, all included trials in this meta-analysis showed concordance between the two groups, and the merged results for all individual studies also revealed no significant difference. In addition to CO_2_ insufflation, respiratory depression caused by conscious sedation may also lead to CO_2_ retention. We also presented the dosage of sedative drugs used. Only Saito *et* al[15] reported a difference when patients were administered midazolam alone; for the other studies[13, 14, 20, 22] there ws no significant between-group difference with respect to dosage of sedative drugs. Moreover, a significant heterogeneity was observed among the studies owing to the use of combination of sedative drugs and the differences with respect to patient’s sedation level.

Until now, two studies[7, 18] have analyzed residual gas; the results bore some similarities to abdominal discomfort caused by rapid absorption into the bloodstream. The study by Takada et al [23] was the first RCT to show that use of CO_2_ insufflation for ESD could reduce the risk of Mallory-Weiss tear[14]; similar results were later reported by Onogi *et* al[17]. The lower incidence of Mallory-Weiss tear in the CO_2_ group was ostensibly due to lower tension of the gastric mucosa as a result of residual gas in the stomach. More RCTs are needed to assess this issue. Four complications were reported in the seven studies included in the present meta-analysis[14, 15, 19–22]. When perforation occurs, the gas in the gastrointestinal tract leaks into the peritoneal cavity. Owing to faster absorption of CO_2_, use of CO_2_ insufflation is expected to lead to lower intra-abdominal pressure in the postoperative period; however, our meta-analysis showed no significant between-group difference in this respect. Contrary to earlier reports, use of CO_2_ insufflation was associated with shorter procedure time than that associated with use of air insufflation. However, more than half of the included studies did not report the experience level of the surgeons, which could have affected the procedure time.

### Limitations

This meta-analysis has several limitations. First, all included studies were from Asia. Second, several studies excluded patients with severe pulmonary disease. Although Takada *et* al[23]recently reported the safety of CO_2_ insufflation during ESD in patients with pulmonary dysfunction under conscious sedation, it was a single-center and uncontrolled study. Other notable limitations of our meta-analysis include heterogeneity among the included studies, inconsistency in the reported outcomes, incomplete data on some of the variables, the relatively small number of included studies and the possibility of publication bias. Some of the outcomes such as abdominal distension are liable to be effected by the depth of sedation and the skills of the endoscopist. Subgroup analyses to assess the effect of these variables could not be performed due to lack of original data. Only two studies reported data on abdominal distension[13, 14]. More robust studies are required to draw definitive conclusions.

## Conclusion

In conclusion, this meta-analysis indicates that CO_2_ insufflation may offer advantages over air insufflation with respect to postoperative abdominal discomfort, procedure time, and the residual gas volume. No significant difference was observed with respect to PtcCO_2_, PaCO_2_, SpO_2_%, abdominal circumference, length of hospital stay, sedation dosage, complications, WBC count, serum CRP level, and dysphagia. More RCTs are required to assess its advantages and necessity in future.

## Supporting information

S1 Table Construction of search strategy. EMBASE: Excerpta Medica Database; Sinomed: Chinese Biomedical Literature Database; MeSH: Medical Subject Heading; ESD: Endoscopic submucosal dissection.
(DOCX)

S2 Table PRISMA 2009 checklist. Preferred reporting items for systematic reviews and meta-analyses.
(DOCX)
