# Supplementary material for: CO2 insufflation versus air insufflation for endoscopic submucosal dissection: A meta-analysis of randomized controlled trials
Source: PLoS One. 2017 May 24;12(5):e0177909. doi: 10.1371/journal.pone.0177909 (PMC5443502; doi:10.1371/journal.pone.0177909)
Supplement: S1 Table — EMBASE: Excerpta Medica Database; Sinomed: Chinese Biomedical Literature Database; MeSH: Medical Subject Heading; ESD: Endoscopic submucosal dissection. (DOCX) [file pone.0177909.s001.docx]

**Supporting Information**

**S1 Table Construction of search strategy**

| **Databases** | **Periodof search** | **Search strategy** |
| --- | --- | --- |
| **Pubmed** | Until January 9, 2017 | ((((((ESD[Title/Abstract]) OR endoscopic submucosal dissection[Title/Abstract]) OR submucosal endoscopy[Title/Abstract])) AND ((((Conventional air[Title/Abstract]) OR Room air[Title/Abstract])) OR "Air"[Mesh])) AND (((((((Dioxide[Title/Abstract]) OR Carbon[Title/Abstract]) OR Carbonic Anhydride[Title/Abstract]) OR Anhydride[Title/Abstract]) OR Carbonic[Title/Abstract])) OR "Carbon Dioxide"[Mesh])) |
| **EMBASE** | Until January 9, 2017 | 1. ‘endoscopic submucosal dissection’/exp OR ‘endoscopic submucosal dissection’ 2. ‘ESD’/exp OR ESD 3. ‘carbon dioxide’/exp OR ‘carbon dioxide’ 4. ‘CO2’/exp OR CO2 5. ‘air’/exp OR ‘air’ 6. ‘room air’/exp OR room air  7. ‘Conventional air’/exp OR Conventional air 8. 1 OR 2 9. 3 OR 4 10. 5 OR 6 OR 7 11. 8 AND 9 AND 10 |
| **The Cochrane library** | Until January 9, 2017 | #1MeSH descriptor: [Carbon Dioxide] explode all trees #2MeSH descriptor: [Air] explode all trees #3Dioxide Carbon:ti,ab,kw (Word variations have been searched) #4Carbonic Anhydride:ti,ab,kw (Word variations have been searched) #5Anhydride, Carbonic:ti,ab,kw (Word variations have been searched) #6Conventional air:ti,ab,kw (Word variations have been searched) #7ROOM AIR:ti,ab,kw (Word variations have been searched) #8"ESD":ti,ab,kw (Word variations have been searched) #9"endoscopic submucosal dissection":ti,ab,kw (Word variations have been searched) #10Submucosal endoscopy:ti,ab,kw (Word variations have been searched) #11 #1or #3 or #4 or #5 #12 #2 or #6 or #17 #13 #8 or #9 or #10 #14 #11 and #12 and #13 |
| **Sinomed** | Until January 9, 2017 | Search terms resemble the terms used in MEDLINE in Chinese. |

EMBASE: Excerpta Medica Database; Sinomed: Chinese Biomedical Literature Database; MeSH: Medical Subject Heading; ESD: Endoscopic submucosal dissection
